# Animal models of the placenta accreta spectrum: current status and further perspectives

**DOI:** 10.3389/fendo.2023.1118168

**Published:** 2023-05-08

**Authors:** Yongdan Ma, Yongyan Hu, Jingmei Ma

**Affiliations:** ^1^ Department of Obstetrics and Gynecology, Peking University First Hospital, Beijing, China; ^2^ Laboratory Animal Center, Peking University First Hospital, Beijing, China; ^3^ Beijing Key Laboratory of Maternal Fetal Medicine of Gestational Diabetes Mellitus, Beijing, China

**Keywords:** placenta accreta spectrum, animal model, placenta development, mouse placenta, trophoblast invasion, decidual deficiency

## Abstract

Placenta accreta spectrum disorder (PAS) is a kind of disease of placentation defined as abnormal trophoblast invasion of part or all of the placenta into the myometrium, even penetrating the uterus. Decidual deficiency, abnormal vascular remodeling in the maternal–fetal interface, and excessive invasion by extravillous trophoblast (EVT) cells contribute to its onset. However, the mechanisms and signaling pathways underlying such phenotypes are not fully understood, partly due to the lack of suitable experimental animal models. Appropriate animal models will facilitate the comprehensive and systematic elucidation of the pathogenesis of PAS. Due to the remarkably similar functional placental villous units and hemochorial placentation to humans, the current animal models of PAS are based on mice. There are various mouse models induced by uterine surgery to simulate different phenotypes of PAS, such as excessive invasion of EVT or immune disturbance at the maternal–fetal interface, which could define the pathological mechanism of PAS from the perspective of the “soil.” Additionally, genetically modified mouse models could be used to study PAS, which is helpful to exploring the pathogenesis of PAS from the perspectives of both “soil” and “seed,” respectively. This review details early placental development in mice, with a focus on the approaches of PAS modeling. Additionally, the strengths, limitations and the applicability of each strategy and further perspectives are summarized to provide the theoretical foundation for researchers to select appropriate animal models for various research purposes. This will help better determine the pathogenesis of PAS and even promote possible therapy.

## 1 Introduction

Placenta accreta spectrum disorders (PAS) refer to a group of diseases in which abnormal trophoblasts invade part or all of the placenta into the myometrium of the uterine wall (1). Although the first case series of placenta accreta (PA) was published in 1937 by Irving and Hertig (2), PAS was a relatively newly defined disorder of placentation until 2018, when the International Federation of Gynecology and Obstetrics (FIGO) named the morbidly adherent placenta, abnormal invasive placenta, adhesive placenta, and other related series of placenta accreta disease as PAS (3). Three subtypes were differentiated from PAS according to the depth of trophoblast invasion into the myometrium (1): placenta accreta, where the villi attach directly to the surface of myometrium without intervening decidua (2); placenta increta, where the invasion of the trophoblast has been into the myometrium over the 1/3; and (3) placenta percreta, where the invasion of villous reaches and penetrates through the myometrium, serosa, and surrounding structures, such as bladder (1, 4). Noticeably, there are often multiple degrees of EVT invasion into the myometrium in one patient, such as the presence of both accreta and percreta in one patient (1). Histopathological examination, the gold standard for the diagnosis of PAS, is very important to detect the occurrence and depth of abnormal EVT invasion (5).

The prevalence of PAS ranged from 0.01% to 1.1% from 1981 to 2012, and continues to increase, especially in countries with high caesarean section (CS) rates (6, 7). Compared with other pregnancy-related diseases, such as gestational diabetes mellitus (GDM) or preeclampsia (PE), the incidence of PAS is relatively lower (8). However, maternal mortality in PAS patients is higher than that in other obstetric diseases due to severe postpartum hemorrhage (4, 9–11). Therefore, it is urgent to clarify the mechanisms of PAS, which will definitely help to improve pregnancy outcomes for patients.

At present, little is known about the pathogenesis of PAS. It is generally accepted that incomplete or absent decidua in pregnancy is the main cause (12). Normal decidualization of the endometrium can prevent excessive invasion of trophoblast cells and suppress the maternal immune response to maintain implantation and decidualization (13). When the structural integrity of the endometrium or myometrium is impaired, scars form locally and the myometrium around the scar often shows degeneration and hyalinosis, accompanied by fibrous tissue hyperplasia and inflammatory cell infiltration, which is not conducive to embryo implantation and placental development (14, 15). When the placenta is attached to the scar site, the villi directly contact the myometrium, which is one of the pathogenic mechanisms of PAS (16). Recently, researchers have suggested that the distorted uteroplacental interface caused by dense fibrous deposition is an essential factor contributing to abnormal placental attachment (2, 17, 18). This challenged the classical concept that placenta accreta is simply due to villous tissue sitting atop the superficial myometrium without interposed decidua.

**Figure 1:**
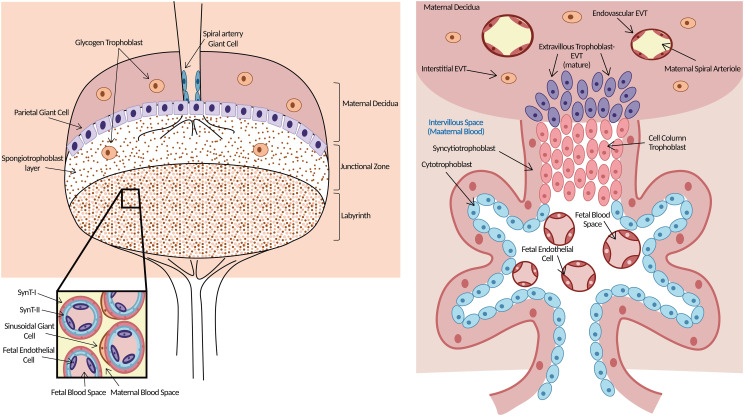
Morphology of mouse (left) and human (right) placenta. Left: Three layers clearly in the mouse placenta. The upper is maternal decidua. The middle layer of the supportive JZ contains spongiotrophoblasts, which give rise to TGCs and glycogen trophoblast cells. Both TGCs and glycogen trophoblast cells are invasive. The labyrinth is the functional structure of the mouse placenta where gas/nutrient exchange occurs. Maternal blood is separated from fetal blood by SynT-I, SynT-II, and sinusoidal giant cells. Right: The functional structure for gas/nutrient exchange is the chorionic villus in the human placenta. EVTs arise from the trophoblast cell column and are highly invasive, penetrating up to one-third of the thickness of the uterus. Endovascular EVTs extensively remodel maternal spiral arterioles to ensure correct blood supply to the growing embryo. Trophoblast giant cells (TGCs), junctional zone (JZ), extravillous trophoblast cells (EVTs), syncytiotrophoblast-I (SynT-I), syncytiotrophoblast- II (SynT-II). Modified from (20).

Elucidating its pathogenesis is the key foundation for improving the pregnancy outcome of PAS patients clinically. Appropriate animal models could facilitate comprehensive and systematic research on the pathogenesis of PAS. Similarities in genetic information between mice and humans have been reported (19). Although the structures of the mouse placenta terminology are highlighted dissimilarity with humans, mouse placentas are highly functionally conserved and the functional placental villous units are remarkably similar in mice and humans (**Figure 1**) (21, 22). In addition, both mouse and human placentas possess similar hemochorial blood flow (20, 23, 24) and undergo trophoblast infiltration and uterine spiral artery remodeling (20, 25–28). Regarding the time of pregnancy period, less time is required in mice, which facilitates animal experiments since it is measured in days rather than in months (27, 28).

Currently, uterine surgeries to simulate the risk factors for PAS are the main approaches to establish disease models. Uterine surgery can damage the endometrium or myometrium, which is helpful to exploring the pathogenesis of PAS from the perspective of “soil” abnormalities. The genetically modified strategy could be another option, which targets the editing of cell invasion or metastasis-related genes; it can not only mimic the abnormal decidua but also simulate the excessive invasion of trophoblast cells or the placenta in PAS, which is helpful for exploring the pathogenesis of PAS from the perspectives of both the “soil” and the “seed” respectively.

This review details early placenta development in mice, with a focus on the approaches of PAS modeling. Additionally, the strengths and limitations of each strategy and further perspectives are summarized to provide the theoretical foundation for researchers to select appropriate animal models for different research purposes. This will help better determine the pathogenesis of PAS and even promote possible therapy.

## 2 Placental development in mice

**Figure 2:**
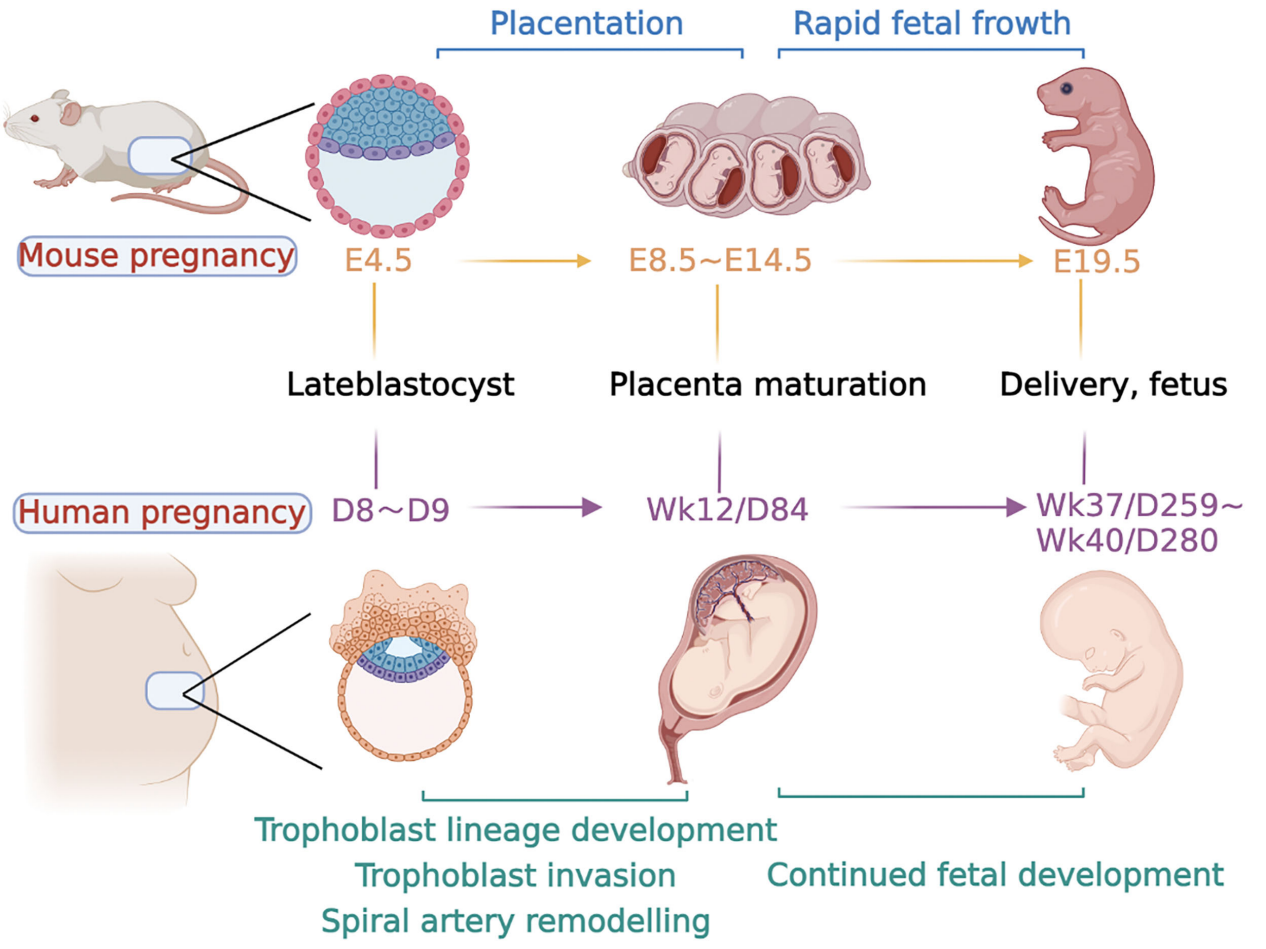
Major event time points in mouse and human pregnancy. The timeline for both species is calculated from conception. Days of development are denoted embryonic (E) in the mouse and week (Wk) or day (D) of development in the human. Late blastocyst implantation in mice occurred at approximately E4.5, followed by endometrial decidualization. The formation of the labyrinth at approximately E8.5, with branching morphogenesis complete by E10.5. At E10.5, the junctional zone and TGCs are also fully formed, and glycogen trophoblast cells continue to differentiate and invade the uterus along with specific TGC subtypes and form a fully functional placenta. At the same time, the embryo developed rapidly at the later stage until delivery at approximately E19.5. In humans, after a series of processes, such as trophoblast cell invasion and uterine spiral artery remodeling, a mature placenta is formed at approximately 12 weeks of gestation. The fetus develops rapidly in the later stage and is normally delivered at approximately 37–40 weeks.

The oocytes and sperm at fertilization to form totipotent zygote are the beginning of embryogenesis in both humans and mice, which occurs at embryonic day (E) 2 in mice. Two distinct populations of cells, the inner cell mass (ICM) and trophoblast ectoderm (TE), emerge through the establishment of cell polarity and several rounds of cell division, respectively, which occurs at E3.5 in mice and approximately day 5 postcoitum in humans to form the blastocyst (**Figure 2**) (19). Blastocyst implantation occurs at E4.5 in mice and days 7–8 postcoitum in humans, when maternal endometrial stromal cells undergo a specific reaction called decidualization under the influence of a variety of hormones, which is widespread in both mice and humans (29). During decidualization, the accurate and ongoing crosstalk between embryonic-derived trophoblast cells and the decidua is an essential “warrantor” for further successful placentation and pregnancy (30). Any factors that lead to impaired decidualization, such as endometriosis or uterine scarring, exert a higher risk of infertility and abnormal placentation (31, 32).

The TE cells in mouse blastocysts continue to proliferate after implantation to form embryonic ectoderm (ExE) and the ectoplacental cone (EPC). At approximately E6.5, the extraembryonic mesoderm lineage is generated, which further forms the allantois and extraembryonic mesodermal layers of the amnion and chorion (19). At approximately E8.5, the fusion of the chorion and allantois is the essential step in the process of forming a mature placenta. Chorion and allantois fusion allows the endoderm-derived blood vessels of the embryo to enter the chorionic trophoblast layer (33). This interfinger differentiation between fetal blood vessels and trophoblast-lined maternal sinuses forms the labyrinth, the basic structure of the mouse placenta (34). After that, the multipotent progenitor cells in the mouse placenta facilitate the expansion of the labyrinth, as well as the differentiation into syncytial trophoblast cells (STB) and sinus trophoblast giant cells (TGCs) (35, 36).

At approximately E10.5, three layers clearly developed in the mouse placenta: the decidua, the junctional zone (JZ), and the labyrinth (20, 37). The labyrinth, which is near the fetal side, is a functional structure where gas–nutrient exchange occurs (38). Maternal blood is separated from fetal blood by three layers of trophoblasts in this layer (39). In the human placenta, there are initially two layers of chorionic villi, and later in pregnancy, there is one functional trophoblast layer that separates maternal and fetal blood (20). The function of the labyrinth is similar to that of the human chorionic villus. The JZ is next to the labyrinth and lies directly beneath the decidua, which consists of parietal trophoblast giant cells and spongiotrophoblasts. Spongiotrophoblasts give rise to TGCs and glycogen trophoblast cells that largely function in hormone secretion and metabolism (22, 40). More importantly, both TGCs and glycogen trophoblast cells are functionally invasive, similar to human EVTs (41), although the two kinds of trophoblast cells anchoring the placenta to the uterine wall in the mouse are not nearly as invasive as the equivalent EVTs in humans, where these cells invade up to one-third of the thickness of the uterine wall, including the maternal arterioles (20). Some glycogen trophoblast cells migrate deep into the maternal decidua, where they are thought to help enhance maternal blood flow (20). All of these properties lie down the foundation for exploring human EVT-related diseases through a focus on TGCs and glycogen trophoblast cells in the mouse placenta.

Although there are some discrepancies in the morphology and terminology of the mouse placenta from the human placenta, the two species appear more equivalent at the functional placental villous units and the cellular composition level. Therefore, the mouse placenta is an appropriate tool for research on human placenta-related diseases.

## 3 Approaches to establish the PAS model

### 3.1 Surgery-induced models

Prenatal prediction of PAS is important to determine the appropriate delivery time and multidisciplinary planning for operative management (42), which is mostly based on ultrasound signs and risk factors (12). The main risk factors for PAS include cesarean section, placenta previa, *in vitro* fertilization and embryo transplantation, and uterine surgery history, such as hysteroscopic operation and endometrial curettage (43–45).

Most of the existing PAS animal models simulate PAS by uterine surgery, such as uterine incision, endometrial curettage, and hysterotomy, and check the pathological phenotypes of placentas in the subsequent pregnancy. Any surgery that damages the endometrium or myometrium may affect the regeneration and repair of endometrial epithelial cells, further resulting in the loss of decidua and the excessive invasion of EVT in the subsequent pregnancy, leading to possible placental abnormalities (46, 47). The PAS models established by different uterine surgeries are described in the following.

#### 3.1.1 Uterine incision

The morphological changes and healing process of the mouse uterine incision within 2 weeks after surgery are similar to humans (48). In terms of the healing process, the epithelium of the lesion of the mouse uterus began to repair on the second day after the operation, and on the fourth day, the wound was completely filled with endometrial tissue on the uterine cavity side and covered by adipose tissue attached to the mesentery on the abdominal side (48). Moreover, the process of healing of the postoperative incision is associated with the estrus cycle of mice, and it is also similar to humans in that the process of healing is associated with changes in hormones (49).

It has been reported that making an incision (0.5–0.8 cm) on one side of the uterine horn of C57BL/6J mice simulates uterine damage, and the placenta showed pathological changes similar to PAS in the subsequent pregnancy (50). Specifically, although the areas of the placental JZ and labyrinth were not significantly different between the model and control groups, nor was the ratio of the placental JZ to labyrinth different, the model group showed a larger area of invasive trophoblast cells in the decidua than that in the control group. This corresponds to the extensively altered expression of genes involved in cellular invasion at the placental interface, such as *Mmp2*, *Mmp9*, *Mmp3*, and *Dock4* (50–52). Meanwhile, the number of blood vessels in the placenta increased significantly in this model, which is similar to the hypervascularity in the placenta of PAS patients (53, 54). In accordance with the hypervascularity in the maternal–fetal interface, the serum levels of the angiogenic factors epidermal growth factor (EGF) and vascular endothelial growth factor (VEGF) tended to increase, while the levels of the antiangiogenic factors soluble fms-like tyrosine kinase 1 (sFlt) and endoglin (ENG) decreased in this model (50). Regarding the immune environment in the decidua and placenta, the proportions of T and natural killer cells (NK) in the decidua diminished significantly at the maternal–fetal interface in this model, and the number of NK cells and M2 macrophages showed a great decline, especially with respect to decidual NK (dNK) cells (50). In terms of inflammatory conditions, the expression of TNF-α and IL-4 in the decidua was upregulated but the expression of IFN-γ and IL-10 was downregulated in this model. These phenotypes are similar to the altered immune environment at the maternal–fetal interface in PAS patients, which is often accompanied by abnormal polarization of macrophages (55) and immune cell infiltrates (56).

In this PAS model, the increased IL-4 may function as a “shooter” to the overactive immune environment at the maternal–fetal interface, which further induces M2-like THP-1 macrophages to increase the expression of granulocyte colony-stimulating factor (G-CSF), prompting the proliferation, invasion, and adhesion of trophoblast cells (55). These alterations are different from PE, where decreased IL-4 in return activates B cells, prompting autoantibody production and eventually leading to endothelial dysfunction and hypertension (57, 58). Compared to the increased IL-4 in this PAS model, the decreased IL-4 in PE is consistent with the notion that shallow cytotrophoblast invasion is implicated in the pathogenesis of PE (58–61). These findings indicate that the immunomodulation in PE and PAS may be totally different, although both are diseases caused by abnormal placental development. The in-depth immune regulation mechanism could also be one of the directions for future research in both PE and PAS.

The uterine incision in the mouse led to an expansive invasion of trophoblast cells, combined with alterations in the immune microenvironment and cytokine levels in the decidua, which recapitulates the classical phenotypes of PAS in the clinic. Therefore, the model can be applied to investigate the abnormal placentation induced by uterine damage, especially to study immune disorders and inflammation regulation at the maternal–fetal interface in PAS, which are currently few studies. In addition to placental abnormalities, changes in serum sFlt and VEGF were found in this model, which suggested that it could be used for the study of serological changes in PAS, further prompting the development of clinical diagnostic markers and prediction models for PAS.

#### 3.1.2 Caesarean section

Caesarean section (CS) is an independent risk factor for PAS (62). It is essential to note that the increase in the incidence of PAS paralleled the promotion of CS (5, 12). The relative risk of PAS was 8.8 (95% CI: 6.1–12.6) for previous CS, 6.6 (95% CI: 4.4–9.8) for a single CS, 7.4 (95% CI: 4.4–9.8) for two CS, and 55.9 (95% CI: 25.0–110.3) for a history of three or more CS (45). The process of uterine tissue repair after CS is often accompanied by decreasing endometrial glands, inhibition of myometrial smooth muscle cell growth, excessive collagen fiber deposition, and massive leukocyte infiltration, which leads to long-term and chronic inflammation and scarring (63). There appears to be preferential attachment of the blastocyst to the scar site, which may be associated with defective decidua in that region, resulting in abnormal implantation (64). In turn, disorientation of the implanted embryo and restricted stromal cell proliferation could also lead to defective decidualization and abnormal placental development, including excessive invasion of trophoblasts into the decidua and insufficient branching of fetal blood vessels (65, 66). Clinically, single-cell RNA sequencing of the PAS placenta of humans shows that in the absence of the decidua, invasive trophoblasts of various differentiation states interact strongly with maternal stromal cells, which might allow the placental villi to migrate to the serosal surface of the uterus and be associated with hypervascularity in PAS patients (67). All of these results indicate that incomplete or absent decidua is one of the key drivers of PAS.

S. D. Burke et al. simulated CS in peripartum mice by hysterotomy in experimental CD-1 mice. Concretely, an incision was made along the entire length of the experimental uterine horn, alongside the mesometrial insertion of blood vessels within 24 h of parturition, which destroyed the integrity of the myometrium and formed the uterine scar locally (68). When the recovered mice were pregnant, an increased depth of trophoblast invasion in the unmanipulated uterine horn was observed, which was assessed by measuring the cytokeratin-positive area extending beyond the placenta proper. This finding indicated that placental paracrine or endocrine signaling may induce a similar placenta accreta phenotype in the unmanipulated horn. In addition, when the researchers examined the disruption or dysplasia of the uterine smooth muscle by HE staining, they found that in this peripartum scar model, the myometrial integrity was significantly disrupted in both the manipulated and unmanipulated uterine horns (68).

This model simulates the characteristics of myometrium damage after clinical CS through hysterectomy in peripartum mice, and the impaired integrity of the myometrium is an important cause of excessive EVT invasion. However, the mechanism and signaling pathway of how myometrial injury affects EVT invasion and placental development is rarely reported. Only a few studies have reported that EVT migration and invasion into the myometrium may be related to the downregulation of functional E-cadherin in the myometrium of uterine scars (64). This model is very suitable for research on the pathomechanism of PAS in the aspect of impaired myometrium. Moreover, it is worth noting that an increase in the depth of trophoblast invasion in the unmanipulated uterine horn was observed in this model, while how the damaged uterus may induce PAS phenotypes in the unmanipulated uterine horn through paracrine or endocrine signaling is also an interesting direction for further investigation. This will help to understand the clinical pathological mechanism of placenta accreta in non-uterine scar sites.

#### 3.1.3 Dilation & curettage

Dilation & curettage (DC) is a surgery generally considered to be relatively safe, but it is still associated with some long-term complications. The most well-known complication is intrauterine adhesions, also known as Asherman syndrome, which may lead to menstrual disorders and fertility problems (69). Previous studies have shown that the uterus of Sprague Dawley (SD) rats presents a series of abnormal changes when intrauterine adhesions occur, such as a decreased number of endometrial glands, increased deposition of collagen and fiber deposition, and disorganized morphology and structure of the myometrium (70, 71). A history of D&C is associated with the occurrence of PAS (OR 2.8; 95% CI 1.7–4.6) (62, 72). However, published studies mainly focus on the assessment of risk factors and the diagnostic utility of D&C in PAS; there is a lack of studies on the mechanism by which D&C affects blastocyst implantation and subsequent placental development.

In this kind of PAS model induced by D&C, three rounds of oblique angle curetting of the endometrium were used to simulate the history of D&C in CD-1 mice. When the recovered mice were pregnant, researchers found that the trophoblast invasion areas were deeper in the experimental uterine horn combined with significant myometrium impairment (68). This strategy to construct the PAS model indicates that D&C can affect the repair of damaged myometrium and subsequent pregnancy outcome. Further studies are clearly needed to address the poorly understood physiology of involution and regeneration of the myometrium after surgery and may help to elucidate the pathogenesis of PAS from the aspect of damaged myometrium.

The above methods to establish PAS models by uterine surgery damaged the normal structure of the endometrium or myometrium of mice, which further facilitated EVT invasion. Moreover, uterine incision surgery can lead to immune imbalance and inflammatory infiltration in the uterine environment, causing local tissue homeostasis imbalance in the uterus, which in turn leads to PAS through multiple immune and inflammation-related signaling pathways (56, 73), which also highlights that PAS is a multifactorial disease. The strengths, limitations, and applicability of each uterine surgery model are shown in **Table 1**. In summary, these approaches comprehensively reflect the pathological phenotype of PAS and are appropriate to explore the pathogenesis of PAS from the perspective of “soil” abnormalities, either the damaged endometrium or myometrium. However, there is a lack of published research to explore the possibility that IVF could be accessible to establish the PAS model, so it is an important direction worth exploring to establish a new model.

**Table 1:** Summary of modeling methods, strengths and limitations, and applicability of the PAS mouse models.

Model	Approaches	Strengths	Limitations	Applicability	Reference
**Surgery-induced models**	Uterine incision	Accompanied by impaired immune and inflammatory environment in the maternal–fetal interface	Invasive surgery, possibly embryonic resorption	Suitable for the research of disordered immune and inflammation in maternal–fetal interface of PAS	(50)
	Cesarean section	Damage to the myometrium, simulate the most essential PAS risk factor CS	Invasive surgery, disturb the estrous cycle	More suitable to investigate the pathomechanism of PAS in the aspect of myometrium abnormalities	(68)
	Dilation & curettage	Surgical procedures before pregnancy, mostly impair the endometrium	Invasive repeated operation	More suitable to investigate the pathomechanism of PAS in the aspect of endometrial abnormalities	(68)
**Genetically modified models**	Simulate the decidual deficiency (Gab3 knockout)	High stability theoretically, non-invasive, pinpoint the role of specific gene in PAS progress	The modeling success rate might be relatively low, possible embryonic death, lower birth rates	Suitable to investigate the pathomechanism of PAS in aspect of decidua deficiency, also to explore the mechanism of uNK cells in the PAS	(74)
	Simulate the excessive invasion of EVT (MARVELD1knockout)			Simulate the excessive invasion of trophoblast cells of PAS	(75)
	Simulate the invasion of the mature placenta (adrenomedullin knockdown)			Optimal tool to study the placental vascular remodeling in PAS, also suitable to develop the angiogenesis-related marker in serum.	(76)

### 3.2 Genetically modified models

With the development of the clustered regularly interspaced short palindromic repeat-associated nuclease 9 (CRISPR−Cas9) and the cyclization recombinase locus of X (cross)-over in P1(Cre-loxP) target genome editing systems, genetically modified mice have been widely used and are considered to be an ideal tool to study molecular and genetic pathways in multiple diseases, especially cancers and some monogenic diseases (77). In addition, genetically modified animal models are also researched in other pregnancy-related diseases, such as GDM, PE and PAS.

Current genetically modified mouse models have been established to mimic the aberrant function of both “soil” and “seed.” Specifically, editing of some targeted genes could result in the abnormality of decidua, and editing of other targeted genes could result in excessive invasion of EVT or the mature placenta. The pathological phenotype and pregnancy outcome of each genetically modified model are depicted in the following sections.

#### 3.2.1 Decidual deficiency

Normal decidua can prevent excessive invasion of trophoblast cells and suppress the maternal immune response to maintain implantation and decidualization (13). Incomplete or absent decidua may be involved in the pathogenesis of PAS (78).

Recently, a GRB2-associated binding protein 3 knockout (Gab3^-/-^) mouse model showed reduced decidual depth in mouse placentas and the spiral artery walls appeared to be heavily invaded by TGCs at approximately E12.5 (74). On E18.5, Gab3^-/-^ mice showed a significant expansion of trophoblasts in the labyrinth and JZ in placentas, leading to an overall increase in the depth of the labyrinth, JZ, and uterine wall (74). GRB2-associated binding (Gab) family proteins, including Gab1–3, are involved in the assembly of intracellular activation signaling complexes by acting as scaffolds to perform docking functions (79, 80). As a regulator, Gab3 is essential for NK cells to perform their functions, including uterine NK (uNK) cells (74). uNK cells are predominantly located in the decidua and are required for the early development of the decidua base and vascular remodeling during pregnancy (81–87). Many studies have shown that uNK cell deficiency facilitates impaired trophoblast invasion in the mouse placenta (88–90). Gab3^-/-^ mice exhibited impaired uNK cell expansion associated with abnormal spiral artery remodeling and increased trophoblast invasion in the decidua basalis (74). After Gab3 knockout, the average minimal distance between trophoblasts and the uterine wall was ~130 mm, whereas the average minimal distance from the wild-type mouse placenta was ~490 mm, which further indicated that Gab3 is a key component required for cytokine-mediated NK-cell priming and expansion that is essential for limiting trophoblast cell invasion during pregnancy (74).

#### 3.2.2 Excessive invasion of EVT

As mentioned earlier, excessive invasion by EVT cells contributes to PAS onset (91). In addition to “soil” abnormalities, EVT invasion can be facilitated. Some genetic mutations related to cell-cycle activity can also lead to changes in the cell’s own ability to invade, which means abnormalities in “seed.” There are some animal models made by editing the genes that regulate the proliferation and invasion of cells to simulate this pathological phenotype of PAS.

In 2018, a genetically modified mouse model of PAS was developed by conditional knockout of MARVEL domain containing 1 (MARVELD1^−/−^), a tumor-suppressor gene located on human chromosome 10q24.2 (92). MARVELD1 has important biological functions in DNA damage repair, cell proliferation, metastasis, and invasion (93). It has been reported that the expression of MARVELD1 is ubiquitous in the endometrium, ovary, and other normal human tissues but is downregulated by promoter methylation in multiple primary tumors derived from the ovary, uterus, breast, testis, and so on (94–97). Yue Chen et al. reported that MARVELD1 was highly expressed in the wild-type mouse placenta, and its expression was increased in the placentas from E10.5 to E18.5, which paralleled ongoing trophoblast cell invasion. On E18.5, MARVELD1 was highly expressed in trophoblast cells that tended to migrate into the JZ (75). For the molecular mechanism, MARVELD1 binds to the integrin β4 promoter to activate its transcription, and active integrin β4 facilitates cell adhesion and suppresses cell migration. In the MARVELD1^−/−^ mouse placenta, the boundary of the JZ was indistinct and was highly occupied by trophoblast cells, combined with the abnormal adhesion of the placenta to the myometrium, which is a placenta accreta phenotype exactly (75). On E18.5, placenta attached to the maternal uterus and the trophoblast cell invasion area into the maternal decidua was increased 3.83 times in MARVELD1^−/−^ mouse placenta compared to the wild type, which was observed by cytokeratin 18 staining (75). All of these results could be explained by the signaling that the deletion of MARVELD1 resulted in decreased expression of integrin β4 in labyrinth layer trophoblast cells, and the adhesive ability of cells was suppressed, which boosted cell migration and invasion, leading to the excessive invasion of trophoblast cells and the placenta accreta phenotype in this model (75).

In regard to cancer and PAS, there are definitely some interesting parallels (98). For example, EVT cells and cancer cells both possess considerable invasion ability and epithelial–mesenchymal transformation (EMT) in the process of migration and invasion (99, 100). Therefore, some cell invasion, metastasis, angiogenesis, and immune-associated genes that have been elucidated in cancer development may be important regulators involved in PAS progression, and it could be one of the directions for research in the future.

#### 3.2.3 Invasion of the placenta

Invasion of fetal placental tissue beyond the maternal decidua causes abnormal anchoring of the placenta to the uterine myometrium, which is the typical ultrasonographic manifestation of PAS patients (101, 102). Invasion of the placenta into the uterus is associated with significant maternal morbidity and mortality due to the possibility of hemorrhage during delivery (103). It has been reported that in adrenomedullin knockout heterozygotes (AM^+/-^), the fetal placental tissue invaded into the adjacent maternal–fetal interface, hypertrophied, and extended beyond the confines of the maternal decidua. H&E staining showed the abnormal invasion of TGCs, a small area of labyrinth layer, a significantly reduced spongiotrophoblast layer, and almost no maternal–fetal interface (76). In other words, the AM^+/-^ intercross placenta completely lacked the normal histological characteristics of a wild-type placenta. All of these characteristics resemble the abnormal invasion of placental tissue into uterine tissue (76). In addition to morphological changes in the placenta, overcrowded conceptuses, a high incidence of fetal growth restriction (FGR) in AM^+/–^ intercrosses, and embryonic loss occurring between E9.5 and E12.5 are the main reasons for the reduced fertility observed in female AM^+/–^ mice (76). This is consistent with the circumstance that except for hemorrhage or other adverse maternal pregnancy outcomes, clinical PAS patients possibly have FGR in some cases (104–106).

Adrenomedullin (AM) is located on human chromosome 11p15.4. The protein encoded by this gene is a preprohormone that is cleaved to form two biologically active peptides, adrenomedullin and proadrenomedullin N-terminal 20 peptide. Adrenomedullin is a bioactive peptide consisting of 52 amino acids (107) that possesses a variety of biological functions, including vasodilation, regulation of hormone secretion, promotion of angiogenesis, and antimicrobial activity (108–111). Previous studies have shown that AM and its receptors are highly expressed in the uterus and placenta, especially in the fetal membranes and umbilical cord blood vessels, which could relax the placental blood vessels in a dose-dependent manner through autocrine or paracrine forms and reduce the resistance of placental blood vessels (112, 113). Abnormal AM levels are related to some pregnancy-related diseases, such as GDM and abortion (114, 115). All of these studies confirmed that adrenomedullin possesses multiple functions in both human pregnancy and animal models (116–118).

In regard to the regulation of placental vascular remodeling and fetal perfusion, in addition to the AM, many molecules have been elucidated to be clearly involved, including VEGF and placental growth factor (PlGF). Correspondingly, there are studies reporting that dysregulating placental vascular remodeling is clearly evident in the majority of PAS cases, which are combined with the alteration of VEGF, PlGF, and their receptors (VEGFR) in both the placenta and serum (119, 120). Therefore, genetically modified animal models based on the current understanding of molecular biological processes involved in the abnormal vascular remodeling of PAS will also become one of the paths to establish new PAS. For example, VEGF-targeted gene editing has been found to be an essential regulator in the placental vascular abnormalities of PAS (73, 120, 121).

Taken together, target gene editing is a non-invasive strategy with good theoretical stability to pinpoint the role of specific genes in disease onset and progression (122). Targeted gene editing in mice leads to abnormal decidua and excessive invasion of trophoblast cells or placenta, which is helpful to exploring the pathogenesis of PAS from both the perspective of “soil” and “seed” abnormalities. However, it should be emphasized that PAS is not a unifactorial disease caused by either uterine or placental abnormalities but is a multifactorial disease. The interactions of “soil” and “seed” lead to PAS. Therefore, when using genetically modified animals, one should not only observe one of the phenotypes but should be explored comprehensively. Additionally, there are still some limitations to genetically modified animals, complete gene knockout mice will lead to embryonic death or birth defects, while the construction cycle of the conditional knockout mouse model is relatively long (123). In addition, the incidence of PAS in targeted gene-edited mice might be relatively low. For instance, 45.95% of MARVELD1^−/−^ mice exhibit the placenta accreta phenotype (75). Researchers should select appropriate target genes and gene editing methods according to the experimental design and objective.

## 4 Summary and prospects

The existing animal models mostly simulate the risk factors or pathological phenotypes by uterine surgeries or targeted gene editing in mice. Various surgeries, whether uterine incision, CS, or D&C, can damage the endometrium or myometrium, which could define the pathological mechanism of PAS from the perspective of “soil.” Genetically modified animal models can not only mimic the abnormal decidua but also simulate the excessive invasion of trophoblast cells or the placenta in PAS, which is helpful for exploring the pathogenesis of PAS from the perspectives of both the “soil” and the “seed” respectively.

Notably, since PAS is a multifactorial disease, it is caused by the interaction between the uterus and placenta. Therefore, the criterion for animal models should not be solely limited to the excessive invasiveness of EVT or the damaged uterus locally. In future studies of animal models, more systematic model evaluation criteria should be established to comprehensively and accurately simulate the clinical manifestations of PAS patients. In addition, when using animal models for research, attention should be given to the difference in the physiological structure of the experimental animal uterus and humans; for example, mice have a bichorned uterus, while surgical modeling is usually performed only on one side, and the unmanipulated uterine horn may be affected by the surgical side through paracrine or endocrine mechanisms. Except for existing genetically modified animal models, other genetically modified animal models based on the current understanding of molecular biological processes associated with PAS will also become one of the paths in the field of PAS animal model development. Undoubtedly, although little is known about the pathogenesis of PAS, the development and application of animal models will help advance the research of PAS to elucidate its pathological mechanism and possible treatment and prevention.

## Author contributions

JM offered the main idea and significant guidance of this manuscript, YM and JM drafted the manuscript, and YH reviewed and revised the manuscript. All listed authors have agreed to the final submitted version.
